# MajesTEC-3: Redefining what’s possible in multiple myeloma

**DOI:** 10.46989/001c.158751

**Published:** 2026-03-12

**Authors:** Samer Al Hadidi, Mahmoud Gaballa

**Affiliations:** 1 Myeloma, Waldenstrom’s, and Amyloidosis Program, Section of Hematologic Malignancies and Cellular Therapy, Simmons Comprehensive Cancer Center, UT Southwestern Medical Center, Dallas, Texas, USA; 2 Department of Lymphoma - Myeloma, Division of Cancer Medicine, MD Anderson Cancer Center, Houston, Texas, USA

**Keywords:** Multiple myeloma, MajesTEC-3, cure, teclistamab, daratumumab

The therapeutic landscape of multiple myeloma (MM) treatment has evolved dramatically over the past two decades. Long-term follow-up data showed that approximately one-third to one-half of patients can achieve survival extending 15 to 20 years from diagnosis when treated with intensive, time-limited regimens.^1^ These findings established proof-of-principle that cure—or at least functional cure—is achievable in MM. The MajesTEC-3 trial has raised the bar even higher, demonstrating that the combination of the BCMA-directed bispecific antibody (BsAb), teclistamab with daratumumab can deliver an excellent 83% reduction in disease progression or death, and a 54% reduction in mortality, compared to standard-of-care daratumumab-based regimens in previously treated patients.^2^

With a median follow-up of 34.5 months, median progression-free survival (PFS) was not reached with teclistamab plus daratumumab versus 18.1 months in the control arm (hazard ratio [HR] = 0.17). The PFS curves appeared to plateau after approximately 6 months, with more than 90% of patients remaining progression-free—a pattern sustained out to 3 years, suggesting the potential for durable disease control. The 36-month overall survival rates were 83% versus 65.0% (HR = 0.46), representing outcomes that rival those seen with BCMA chimeric antigen receptor (CAR) T-cell therapy, but with easier logistics and an off-the-shelf availability.

## The Infection Challenge and a Path Forward

Despite these remarkable efficacy results, the safety profile warrants careful consideration. Grade 3 or 4 infections occurred in 54% of patients receiving teclistamab plus daratumumab, with 13 infection-related deaths. However, this challenge appears modifiable. The infection rates were highest during the first 6 months of treatment, before the implementation of comprehensive prevention guidelines. Following protocol amendments mandating immunoglobulin replacement therapy and antimicrobial prophylaxis, the infection incidence declined substantially. This pattern mirrors the learning curve observed with CAR T-cell therapy, where standardized management protocols for cytokine release syndrome (CRS) and immune effector cell-associated neurotoxicity syndrome (ICANS) dramatically improved safety outcomes.^3–5^ In MajesTEC-3, all cases of CRS were grade 1 or 2 and all resolved, while ICANS occurred in only 1% of patients.

The immunosuppressive nature of MM itself compounds infection risk. Patients with relapsed disease often have compromised immune function from both their underlying malignancy and cumulative treatment exposures. Emerging data suggest that fixed-duration approaches to BsAb therapy may be feasible for patients achieving deep remissions, potentially reducing long-term immunosuppression, while maintaining disease control.^6^ Importantly, a biologically more rational approach may be response-adapted treatment duration guided by minimal residual disease (MRD) assessment, rather than a uniform predefined fixed duration. Under this paradigm, patients who achieve sustained MRD negativity—confirmed by sensitive techniques such as next-generation sequencing or flow cytometry—could be candidates for treatment discontinuation earlier, while those with persistent MRD positivity may benefit from continued therapy for a longer duration. MRD-guided de-escalation strategies would allow individualized treatment decisions, potentially sparing patients from unnecessary prolonged immunosuppression, while identifying those at higher risk for early relapse who require more sustained treatment exposure.

## Treatment Sequencing and the CAR T Question

A critical consideration in interpreting MajesTEC-3 is that only 5% of enrolled patients had prior daratumumab exposure, and none were daratumumab-refractory—an increasingly rare population in contemporary practice, particularly after the PERSEUS trial established daratumumab-based quadruplet therapy as the frontline standard of care.^7^ It is important to note, however, that in PERSEUS, daratumumab maintenance may be discontinued after two years in patients who achieve sustained MRD negativity for at least one year. Consequently, a meaningful proportion of patients relapsing after PERSEUS-based frontline therapy will not be daratumumab-refractory at the time of first relapse, but rather daratumumab-sensitive and lenalidomide-refractory. Accordingly, the magnitude of benefit observed in MajesTEC-3 may not fully reflect outcomes in more daratumumab-exposed real-world populations. MajesTEC-9 directly addresses this limitation by evaluating teclistamab monotherapy in a predominantly daratumumab-refractory population. In this Phase 3 trial of patients who had received 1-3 prior lines of therapy, 85% were refractory to anti-CD38 monoclonal antibodies and 79% to lenalidomide, with more than 90% refractory to their last line of therapy. Despite this high-risk profile, teclistamab monotherapy showed a 71% reduction in the risk of disease progression or death (HR = 0.29; 95% CI: 0.23, 0.38) and a 40% reduction in the risk of death (HR = 0.60; 95% CI: 0.43, 0.83), compared to standard of care. Together, MajesTEC-3 and MajesTEC-9 establish teclistamab-based regimens across the continuum of relapsed MM—demonstrating efficacy in both anti-CD38 sensitive patients (where combination with daratumumab may be optimal) and anti-CD38 refractory patients (where monotherapy retains substantial activity).

The solution may lie in transitioning BsAbs to time-limited treatment paradigms. Rather than continuing BsAbs indefinitely, future trials should evaluate fixed-duration approaches—perhaps 12 to 24 months—for patients achieving minimal residual disease negativity. This strategy would preserve treatment options, reduce cumulative toxicity and infection risk, and potentially improve quality of life. Such an approach mirrors successful strategies in other hematologic malignancies, including chronic myeloid leukemia, where treatment-free remission has become an achievable goal for selected patients. Indeed, the field of MM must embrace time-limited therapy as the standard paradigm moving forward. The era of indefinite treatment should give way to response-adapted approaches that allow patients treatment-free intervals, preserve immune function for subsequent therapies if needed, and restore quality of life without compromising long-term outcomes.

## Equitable Access and Real-World Implementation

The promise of BsAbs extends beyond their clinical efficacy to their potential for democratizing access to cutting-edge therapy. Unlike CAR T-cell therapy, which requires specialized manufacturing facilities and experienced centers, BsAbs can be administered in community settings with appropriate support. However, this potential will only be realized through deliberate efforts to ensure equitable access. Historical disparities in MM treatment access and outcomes persist, and BsAbs clinical trial access disparities were previously reported.^8^

Successful implementation will require investment in community infrastructure, including access to supportive care resources, close monitoring capabilities, and established protocols for managing toxicities. Training programs for community oncologists must address the unique challenges of BsAbs therapy, including early recognition and management of CRS and ICANS, appropriate infection prophylaxis, and patient selection. Additionally, financial toxicity cannot be ignored—cost-effectiveness analyses will be essential to ensure sustainable access to these transformative but expensive therapies worldwide.

## Future Directions

Several key questions must be addressed to optimize the use of teclistamab plus daratumumab and similar combinations. First, can we identify biomarkers that predict which patients will achieve durable remissions with fixed-duration therapy versus those requiring continuous treatment? Second, do we even need to think about sequencing BsAbs, CAR T-cell therapy, and other novel immunotherapies, or can we achieve such durable responses that sequencing becomes irrelevant for many patients? Third, how can we minimize infection risk while maintaining efficacy, perhaps through more aggressive prophylaxis, immunoglobulin replacement, or less intense dosing once complete remission is achieved?

Ongoing trials are already beginning to address these questions. Studies evaluating BsAbs in earlier treatment lines—including newly diagnosed patients—will determine whether the remarkable efficacy seen in MajesTEC-3 can be recapitulated in less heavily pretreated populations. Beyond MajesTEC-9, several other ongoing trials are evaluating bispecific antibodies in earlier lines of therapy, including frontline combinations with anti-CD38 antibodies and immunomodulatory agents, dual bispecific antibody combinations targeting different antigens, and bispecific-based regimens in newly diagnosed transplant-ineligible patients. Collectively, these studies are reshaping the treatment paradigm and will critically inform sequencing strategies in a landscape where frontline exposure to anti-CD38 antibodies and lenalidomide is increasingly the norm. Combination strategies pairing BsAbs with novel agents may further improve outcomes. And, critically, trials incorporating treatment-free intervals or response-adapted approaches will inform whether continuous therapy is truly necessary. These studies must prioritize time-limited treatment endpoints, as the ability to safely discontinue therapy after achieving deep remissions would represent a fundamental shift in how we approach MM care—transforming it from a chronic disease requiring lifelong treatment to one where cure or prolonged treatment-free survival becomes the expectation rather than the exception.

The MajesTEC-3 trial represents a landmark achievement in MM therapeutics, joining the growing body of evidence that long-term disease control and potentially, cure, is within reach for many patients. As we build on these remarkable results, our focus must expand beyond simply demonstrating efficacy to ensuring that these advances translate into improved outcomes for all patients. This will require addressing infection management, optimizing treatment sequencing, transitioning to time-limited therapy where appropriate, and ensuring equitable access. The unprecedented hazard ratio of 0.17 in MajesTEC-3 is not just a statistical achievement—it represents renewed hope for patients and families facing this disease. Our collective responsibility is to transform that hope into reality through thoughtful implementation, continued innovation, and further commitment to health equity.

### Competing interests

**S.A.H.** reports receiving consulting fees from Janssen, Sanofi, and Legend Biotech.

**M.G.** reports receiving consulting fees from Bristol-Myers Squibb, Arcellx and Guidepoint

### Authors’ Contribution per CRediT

Conceptualization: Samer Al Hadidi

Formal analysis and investigation: Samer Al Hadidi, Mahmoud Gaballa

Writing - original draft preparation: Samer Al Hadidi

Writing - review and editing: Samer Al Hadidi, Mahmoud Gaballa
